# Prevalence, Awareness, Medication, Control, and Risk Factors Associated with Hypertension in Bai Ethnic Group in Rural China: The Yunnan Minority Eye Study

**DOI:** 10.1371/journal.pone.0070886

**Published:** 2013-08-12

**Authors:** Jinman Zhang, Qin Huang, Minbin Yu, Xueping Cha, Jun Li, Yuansheng Yuan, Tao Wei, Hua Zhong

**Affiliations:** 1 The First Affiliated Hospital of Kunming Medical University, Kunming, China; 2 The First Hospital of Yunnan Province, Kunming, China; 3 Kunming Medical University, Kunming, China; 4 State Key Laboratory of Ophthalmology, Zhongshan Ophthalmic Center, Sun Yat-sen University, Guangzhou, China; Zhongshan Ophthalmic Center, China

## Abstract

**Purpose:**

This study aimed to assess the prevalence, awareness, treatment, and control of hypertension and their associated factors among Bai ethnic population in the rural China.

**Methods:**

A population-based survey was conducted in 2010 with a randomly cluster sampling in rural communities in Dali, southwest China. A total of 2133 adults aged 50 or above were interviewed, and their blood pressure, height, weight and waist circumference were measured. Hypertension was defined as a mean SBP≥140 mmHg and/or DBP≥90 mmHg, and/or current use of antihypertensive medications.

**Results:**

The prevalence of hypertension was 42.1% (899/2133), and the age- and gender-adjusted prevalence was 40.0%. Among the hypertensive participants, 28.4% (255/899)were aware of their condition, while 24.6% (221/899) took antihypertensive medications, with only 7.5% (67/899) of those achieving blood pressure control (<140/90 mmHg). Risk factors for hypertension were older age, smoking, alcohol drinking, family history of HBP, overweight, and obesity, while protective factors included being lean, and having finished senior high school or above.

**Conclusions:**

Hypertension prevalence is high among the population of Bai ethnic group in China, while the associated risk factors of hypertension include overweight/obesity, cigarette smoking, history of hypertension, and older age. The percentages of hypertensive participants aware of their hypertension and those taking antihypertensive medications were low with an incredibly low proportion of hypertensive patients who kept their hypertension under control. It is suggested that health education and hypertension screening programs be carried out in the area for the high blood pressure prevention and control.

## Introduction

Cardiovascular diseases (CVD) have become one of the major causes of death and disability among the elderly in rural areas in China, with stroke accounting for over 40% of deaths [1]. The prevalence of hypertension, which is one of the key risk factors of CVD, has gone up dramatically [2]. According to the China National Nutrition and Health Survey 2002, among the 24% hypertensive participants who were aware of their condition, 78% took medications and 19% were properly controlled. Meanwhile, the ratio of controlled to treated hypertension was 1:4, similar to an estimate about 20 years ago in 1991 [3].

As shown in previous studies, hypertension prevalence rates increased with age [3], [4], [5]. In addition, hypertension prevalence rates were observed high in some rural areas in China, which were 30.6% [6], 36.09% [4], respectively. According to a study by Yang and colleagues, the prevalence of hypertension in the rural areas in 1991, 2002, and 2007 was 20.4, 24.5 and 30.6%, respectively [6], showing an increase trend. Therefore, the elderly in rural areas have been a concern in the academic community.

However, only a few studies on hypertensionof ethnic groups in Yunnan China, which borders Myanmar, Laos, and Viet Nam, have been conducted. Some of these studies focused on relationship of gene and hypertension [7], and data on hypertension related issues among Bai ethnic group are not available. Most of Bai people live in the rural Dali [8], Yunan, and they have their own unique cultures which are different from those in other ethnic groups. They had created a powerful nation for hundred years in their history [9], [10]. Archaeological finds show that the Erhai area was inhabited as early as the Neolithic Age. The Bai ethnic have their own language derived from the Zang-Mian Austronesian family of Sino-Tibetan Phylum. The Bai area is crisscrossed with rivers. The area around Lake Erhai in the autonomous prefecture has a mild climate and fertile land yielding two crops a year. Therefore, the Bai people mainly are occupied with agriculture. They like eating pickled food and peculiar cheese. As a whole, the Bai ethnic group and the majority of Chinese ethnic groups differ in genetic origin, inhabited environment, economy, and customs. In order to investigate the prevalence, awareness, medication, and control of hypertension among the Bai ethnic group in Dali city (a county-level city) of southwest China, which is the hometown of Bai people, we conducted an investigation on the above-mentioned topics. Totally 2133 people participated in the survey, whose ages were 50 years old or over. Among them, there were 769 men (36.1%) and 1364 women (63.9%).

## Methods

Ethical approval was obtained from the Kunming Medical University Ethics Review Board. The study was conducted in accordance with the tenets of the World Medical Association's Declaration of Helsinki. All participants were included only after they had signed an informed consent form.

Study subjects were enrolled in a randomly selected sample of individuals in Dali, which is one of twelve counties in Dali Bai Nationality Autonomous Prefecture. The decision to select this district for the survey was taken because more than 80% of the population of Bai Nationality in China live in Dali Prefecture and it is a socioeconomic profile representative of Bai Nationality as a whole.

The sampling frame was constructed using geographically defined clusters based on village register data. Cluster boundaries were defined such that each cluster would have a population of approximately 1000 individuals at all ages. Cluster sampling was used to divide Dali County into 259 areas. Sample size was based on estimating an anticipated 3.5% prevalence for glaucoma with 95% confidence interval. Because the prevalence of hypertension was much higher than glaucoma in previous studies, the sample size should be enough. Assuming an examination response rate of 80%, and a design effect of 1.25 to account for inefficiencies associated with the cluster sampling design, a sample of 2118 persons ≥50 years of age was required. Depending on the percentage of population ≥50 years of age, 12 to 14 clusters were randomly selected (with equal probability) by specialists of Zhongshan Ophthalmic Center.

Field work was carried out over 4 months' period, beginning in January 2010. Listing of households with the names of residents ≥50 years of age were obtained from the village registers, followed by door-to-door household visits conducted by enumeration teams. Those ≥50 years of age were enumerated by name, gender, age, and education. Individuals temporarily absent at the time of the household visit were included in the enumeration. Unregistered adults ≥50 years of age were enumerated and included in the study sample if they had been living in the household for 6 months or more.

An examination site was set up in the local community facilities within fifteen minutes' walking distance for most subjects. Study participants were examined on a prescheduled date established at the time of enumeration. Identity of the subjects was verified using the subjects' official photo identity cards. Those who did not appear at the examination site were revisited, repeatedly if necessary, by a member of the enumeration team to encourage participation. Details of the study design, sampling plan, and baseline data are reported elsewhere [11], [12], [13].

A standard questionnaire was administered by a trained interviewer to collect details of hypertension history, general medical history, alcohol drinking, cigarette smoking, and education. We revised the categories of cigarette smoking (“occasional smoking” was merged into “no smoking”, and “quitting smoking” into smoking) and education (“junior college” and “graduate or above” were merged into “junior college and above”) because there were few participants belonging to the above mentioned subcategories merged.

Three blood pressure (BP) measurements were obtained from each participant by trained and certified observers according to a common protocol adapted from procedures recommended by the American Heart Association [14].

BP was measured three times with the participant in the sitting position after 5 min of rest, and the time interval between three measurements of BP was 2 min. In addition, participants were advised to avoid alcohol, cigarette smoking, coffee/tea, and exercise for at least 30 min before their BP measurement. A standardized mercuric-column sphygmomanometer was used, and one of four cuff sizes (pediatric, regular adult, large, or thigh) was chosen based on the circumference of the participant's arm.

All study investigators and staff members successfully completed a training program that oriented them both to the aims of the study and to the specific tools and methodologies employed. At the training sessions, interviewers were given detailed instructions on administration of the study questionnaire. All BP observers participated in a special training session on the use of a standardized protocol for measurement of BP. To be certified as a BP observer satisfactory performance on a written exam testing knowledge of preparing study participants for measuring BP, selecting correct cuff size, and using standard techniques for BP measurement was required. In addition, using standard techniques for BP measurement during a standardized videotaped examination, and during concordant measurements with an instructor also was required.

The hypertension status was assessed based on the US Seventh Joint National Committee (JNC) report on the prevention, detection, evaluation, and treatment of high BP [15].

Hypertension was defined as an average SBP at least 140 mmHg, or an average DBP at least 90 mmHg. Patients who self-reported current treatment for hypertension with antihypertensive medication within the 2 weeks prior to the interview were also classified as hypertensive patients. This definition excludes hypertensive patients whose BP has been reduced to a nonhypertensive range solely by the use of nonpharmacological measures. Self-reported hypertensive medication use is regarded as reliable, as it was based on the participants' medical records, the prescriptions by physicians, and medication boxes brought along with them during the study. The awareness of hypertension was defined by any self-report of any prior diagnosis of hypertension by a healthcare professional as having hypertension. The treatment of hypertension was defined as the selfreported use of pharmacological medication for the management of high blood pressure (HBP) within the 2 weeks preceding the participant's interview. The control of hypertension was defined as the pharmacological treatment of hypertension with an average SBP less than 140 mmHg and an average DBP less than 90 mmHg.

During the health visit, height, weight and waist circumference were measured using a standardized protocol and body mass index (BMI) was calculated in kg/m2. Lean, normal weight, overweight, and obesity were defined as a BMI <18.50, 18.5–24.99, 25.00–29.99, and ≥30.00 kg/m2, respectively [16]. Information on demographics (i.e., age, sex and residential area), socio-economic status (i.e., education level achieved, occupation and annual household income), and the like was collected with a standardized questionnaire that has been used for other studies in China [17], [18].

Continuous variables were given as the mean ± SD and categorical variables as the percentage in each subgroup. Continuous variables between two groups were compared by using t test, while those among three groups or more by using one-way ANOVA. The associations between categorical variables were tested by the use of contingency tables and the Chi-square test. We calculated adjusted odds ratios (ORs) with 95% confidence intervals (95% CIs) for hypertension status by using multivariate logistic regression models. All data analyses were conducted using SAS software (Version 9.1; SAS Institute Inc., Cary, North Carolina, USA). All statistical tests were two-tailed, and P values less than 0.05 were considered statistically significant.

## Results

Overall, 42.1% (899/2133; 95%CI: 40.0, 44.3%) of the adults aged 50 or more among the target population suffered from hypertension, 41.7% (321/769; 95%CI: 38.2, 45.3%) in men and 42.4% (578/1364; 95%CI: 39.7, 45.1%) in women without a statistical difference between them (p>0.05). The age- and gender-adjusted prevalence of hypertension was 40.0% (Table 1).

**Table 1:** Prevalence of hypertension among Bai people aged 50 or more in Dali, southwest China.

	No. of participants	No. of participants detected as hypertensive	Prevalence
Male	769	321	41.7%
Female	1364	578	42.4%
Total	2133	899	42.1%

The values of SBP and DBP between males and females were not statistically different (p>0.05). However, the value of SBP among males (143.57±28.36) showed higher than that among females (138.71±24.79) in the age group 70–79 (p<0.05). The value of SBP in the age group 50–59 (131.86±24.91) showed lower than those among other age groups (age group 60–69: 135.06±24.92, age group 70–79: 140.59±26.31, age group 80 and more: 140.10±27.14,)(p<0.05).

Also performed were gender- and age-specific estimates of the distribution of BP according to the classification system recommended by the Joint National Committee on Detection, Evaluation, and Treatment of High Blood Pressure [14]. In total, 38.2% of men and 37.5% of women had optimal or normal BP (SBP<130 mmHg and DBP<85 mmHg), while 20.0% of men and 20.1% of women had high-normal BP. The prevalence of stages 1, 2, and 3 hypertension was 22.6, 12.1, and 7.0% in men and 23.3, 12.8, and 6.3% in women, respectively.

Totally 28.4% (255/899; 95%CI: 25.4, 31.5%) of the participants with hypertension were aware of their condition, 27.4% (88/321; 95%CI: 22.6, 32.6%) in men, and 28.9% (167/578; 95%CI: 25.2, 32.8%) in women. Of the hypertension participants, 24.6% (221/899; 95%CI: 21.8, 27.6%) took antihypertensive medications, whereas 7.5% (67/899; 95%CI: 5.8, 9.4%) of them turned out to be normotensive with the medications. Among those who took medicines for their hypertension, 30.3%(67/221; 95%CI: 24.3,36.9%) were successful to control their high blood pressures. No statistical differences were observed in awareness, treatment or control by gender or by age groups(Chi-square P>0.05).

According to the results of the Chi square test (Table 2), factors which modified the percentage of HBP in the participants include age group, education level, BMI, cigarette smoking, alcohol drinking, and family history of HBP. People with higher BMI values, older ages, lower levels of education, and a family history of hypertension, had a greater chance of developing hypertension than those without. So did those who smoked, or drank alcohol (compared to people who did not smoke, or did not drink alcohol).

**Table 2:** Percentage (%) of awareness, treatment and control of hypertension by socio-demographics and those with and without risk factors.

Variables	Hypertension (n = 2133)^a^	Awareness (n = 899)^b^	Treatment (n = 899)^b^	Control (n = 221)^c^
Age groups (yrs): 50–59 (reference)	34.6	31.5	27.8	21.7
60–69	41.5*	30.4	26.4	34.1
70–79	51.0*	25.5	21.7	34.5
80–	52.5*	17.7	14.5	33.3
Gender: Men (reference)	41.7	27.4	23.7	28.9
Women	42.4	28.9	25.1	31.0
Education level: No school (reference)	46.0	27.2	22.3	34.6
Primary school	42.2	19.4	19.4	25.0
Junior high school	42.4	29.2	26.2	27.5
Senior high school	36.6*	31.2	26.1	26.8
Junior college and above	32.9	38.5	38.5	40.0
Body weight: normal (reference)	41.6	26.8	23.3	30.1
Leanness	34.8*	12.5*	10.7*	25.0
Overweight	50.7*	43.1*	36.6*	32.1
Obesity	61.8*	52.4*	47.6*	30.0
Cigarette smoking: no (reference)	40.5	29.7	26.3	29.3
Smoking	46.7*	25.1	20.5	31.5
Alcohol consumption: no (reference)	39.6	26.0	22.9	28.9
Yes	54.1*	36.4*	30.1*	33.9
Family history of HBP: no (reference)	31.9	27.3	23.9	33.9
Yes	74.7*	33.8	28.3	24.4

a Among all the participants, ^b^ Among participants with hypertension, ^c^ Among participants taking medications for their hypertension. * P<0.005 in education level (P<0.05/10), P<0.0083 in the body weight group and age group (P<0.05/6), P<0.05 in cigarette smoking group, alcohol consuming group and family history of HBP (compared with the reference by Chi-square test).

Compared to the participants with normal weight, those with their BMI values less than 18.0 appeared less likely to be aware of their HBP, while those regarded as overweight or obesity were more likely to know their hypertensive condition. Subjects who consumed alcohol or smoked were more likely to know their hypertensive condition than those who did not drink alcohol or did not smoke.

In order to further analyze risk factors of hypertension, hypertension was taken as dependant variable, while age group, BMI, education level, diabetes mellitus, cigarette smoking, alcohol consumption, and family history of HBP as independent variables. Multivariate logistic regression models were performed (Table 3), with adjustment for age as well as for all other variables. Meanwhile, age-stratified multivariable analyses were also performed (Table 4, 5, 6, 7) with adjustment for gender and for all other variables, respectively. As a result, the participants with a higher age group level appeared to have a higher risk of developing hypertension (OR>1.0), as is shown in the age group 60–69, 70–79, and 80+, compared with the age group 50–59. Overweight and obese participants were more likely to suffer from hypertension than normal-weight ones (OR>1.0), whereas lean people were less likely to have HBP (OR<1.0). In addition, hypertensive patients with a family history of HBP, or those who drank, smoked, had a higher chance of developing hypertension than those without, or those who did not.

**Table 3:** Factors associated with the prevalence of hypertension from multivariate logistic regression models (N = 2133).

Variables	n	Prevalence of HBP (%)	OR (95% CI)^a^	OR (95% CI)^b^
Age groups (years): 50–59	716	34.6	–	1.000(reference)
60–69	775	41.5	–	1.365(1.080, 1.725)
70–79	524	51.0	–	1.925(1.465, 2.529)
80–	118	52.5	–	2.248(1.432, 3.527)
Body weight: normal weight	1475	41.6	1.000 (reference)	1.000(reference)
Leanness	322	34.8	0.616(0.474, 0.801)	0.556(0.417, 0.741)
Overweight	302	50.7	1.577(1.225, 2.031)	1.591(1.211, 2.091)
Obesity	34	61.8	2.465(1.212, 5.011)	3.023(1.443, 6.334)
Education level: No school	759	46.0	1.000 (reference)	1.000(reference)
Primary school	147	42.2	0.934(0.651, 1.341)	0.909(0.614, 1.346)
Junior high school	719	42.4	0.998(0.805, 1.238)	0.925(0.726, 1.178)
Senior high school	429	36.6	0.853(0.659, 1.104)	0.673(0.501, 0.905)
Junior college and above	79	32.9	0.681(0.414, 1.119)	0.538(0.308, 0.938)
Diabetes mellitus: no	2089	42.0	1.000 (reference)	1.000(reference)
Yes	44	50.0	1.490(0.816, 2.723)	1.427(0.740, 2.754)
Cigarette smoking: no	1570	40.5	1.000 (reference)	1.000(reference)
Smoking	563	46.7	1.246(1.024, 1.515)	1.415(1.128, 1.774)
Alcohol consumption: no	1770	40.2	1.000 (reference)	1.000(reference)
Yes	363	51.8	1.603(1.275, 2.016)	1.656(1.290, 2.125)
Family history of HBP: no	1623	31.9	1.000 (reference)	1.000(reference)
Yes	510	74.7	6.218(4.956, 7.802)	6.516(5.165, 8.219)

95%CI, 95% confidence interval; HBP, high blood pressure; OR, odds ratio;

a Adjusted for age only; ^b^ Adjusted for all other variables in the table.

**Table 4:** Factors associated with the prevalence of hypertension from multivariate logistic regression models in age group 50–59 (N = 716).

Variables	n	Prevalence of HBP (%)	OR (95% CI)^a^	OR (95% CI)^b^
Gender: Female	496	35.9	–	1.000(reference)
Male	220	31.8	–	0.694(0.384, 1.252)
Body weight: normal weight	503	30.6	1.000 (reference)	1.000(reference)
Leanness	58	27.6	0.869(0.474, 1.593)	0.933(0.485, 1.793)
Overweight	139	47.5	2.029(1.382, 2.979)	1.940(1.278, 2.943)
Obesity	16	75.0	6.807(2.160, 21.451)	7.451(2.260, 24.564)
Education level: No school	164	34.1	1.000 (reference)	1.000(reference)
Primary school	44	38.6	1.248(0.626, 2.489)	1.292(0.608, 2.742)
Junior high school	243	36.6	1.152(0.756, 1.757)	1.162(0.731, 1.847)
Senior high school	227	33.0	1.021(0.649, 1.607)	0.979(0.590, 1.622)
Junior college and above	38	28.9	0.861(0.387, 1.916)	0.904(0.373, 2.190)
Diabetes mellitus: no	697	33.9	1.000 (reference)	1.000(reference)
Yes	19	63.2	3.398(1.319, 8.757)	2.931(1.038, 8.277)
Cigarette smoking: no	558	34.8	1.000 (reference)	1.000(reference)
Smoking	158	34.2	1.301(0.747, 2.265)	1.434(0.774, 2.657)
Alcohol consumption: no	599	33.4	1.000 (reference)	1.000(reference)
Yes	117	41.0	1.389(0.925, 2.084)	1.314(0.828, 2.087)
Family history of HBP: no	564	25.7	1.000 (reference)	1.000(reference)
Yes	152	67.8	6.066(4.110, 8.954)	6.235(4.181, 9.298)

95%CI, 95% confidence interval; HBP, high blood pressure; OR, odds ratio;

a Adjusted for gender only; ^b^ Adjusted for all other variables in the table.

**Table 5:** Factors associated with the prevalence of hypertension from multivariate logistic regression models in age group 60–69 (N = 775).

Variables	n	Prevalence of HBP (%)	OR (95% CI)^a^	OR (95% CI)^b^
Gender: Female	468	43.2	–	1.000(reference)
Male	307	39.1	–	0.733(0.451, 1.190)
Body weight: normal weight	569	42.0	1.000 (reference)	1.000(reference)
Leanness	94	26.6	0.496(0.305, 0.808)	0.446(0.262, 0.758)
Overweight	102	52.0	1.475(0.966, 2.253)	1.342(0.840, 2.143)
Obesity	10	50.0	1.356(0.388, 4.743)	1.607(0.417, 6.190)
Education level: No school	214	43.9	1.000 (reference)	1.000(reference)
Primary school	55	47.3	1.169(0.643, 2.126)	1.271(0.671, 2.406)
Junior high school	327	41.6	0.938(0.654, 1.346)	0.876(0.590, 1.301)
Senior high school	156	37.8	0.815(0.521, 1.273)	0.730(0.447, 1.193)
Junior college and above	23	30.4	0.594(0.230, 1.531)	0.548(0.198, 1.516)
Diabetes mellitus: no	759	41.6	1.000 (reference)	1.000(reference)
Yes	16	37.5	0.820(0.294, 2.283)	0.974(0.323, 2.938)
Cigarette smoking: no	557	40.9	1.000 (reference)	1.000(reference)
Smoking	218	43.1	1.610(1.003, 2.585)	1.487(0.888, 2.490)
Alcohol consumption: no	640	39.8	1.000 (reference)	1.000(reference)
Yes	135	49.6	1.502(1.034, 2.182)	1.527(1.016, 2.295)
Family history of HBP: no	601	31.8	1.000 (reference)	1.000(reference)
Yes	174	75.3	6.514(4.432, 9.574)	6.813(4.582, 10.131)

95%CI, 95% confidence interval; HBP, high blood pressure; OR, odds ratio;

a Adjusted for gender only; ^b^ Adjusted for all other variables in the table.

**Table 6:** Factors associated with the prevalence of hypertension from multivariate logistic regression models in age group 70–79 (N = 524).

Variables	n	Prevalence of HBP (%)	OR (95% CI)^a^	OR (95% CI)^b^
Gender: Female	321	49.5	–	1.000(reference)
Male	203	53.2	–	0.618(0.335, 1.141)
Body weight: normal weight	338	54.7	1.000 (reference)	1.000(reference)
Leanness	127	38.6	0.522(0.344, 0.792)	0.373(0.226, 0.614)
Overweight	54	57.4	1.129(0.631, 2.020)	1.662(0.877, 3.150)
Obesity	5	40.0	0.551(0.091, 3.346)	1.265(0.194, 8.259)
Education level: No school	305	53.1	1.000 (reference)	1.000(reference)
Primary school	39	33.3	0.379(0.182, 0.787)	0.315(0.136, 0.732)
Junior high school	126	53.2	0.859(0.544, 1.358)	0.992(0.585, 1.685)
Senior high school	39	48.7	0.674(0.328, 1.385)	0.483(0.211, 1.109)
Junior college and above	15	40.0	0.486(0.164, 1.440)	0.344(0.100, 1.188)
Diabetes mellitus: no	516	51.0	1.000 (reference)	1.000(reference)
Yes	8	50.0	0.982(0.243, 3.974)	0.539(0.111, 2.616)
Cigarette smoking: no	370	46.5	1.000 (reference)	1.000(reference)
Smoking	154	61.7	2.531(1.497, 4.280)	3.815(2.065, 7.050)
Alcohol consumption: no	432	48.6	1.000 (reference)	1.000(reference)
Yes	92	62.0	1.737(1.094, 2.757)	1.708(1.006, 2.901)
Family history of HBP: no	374	38.5	1.000 (reference)	1.000(reference)
Yes	150	82.0	7.252(4.551, 11.556)	10.143(6.025, 17.077)

95%CI, 95% confidence interval; HBP, high blood pressure; OR, odds ratio;

a Adjusted for gender only; ^b^ Adjusted for all other variables in the table.

**Table 7:** Factors associated with the prevalence of hypertension from multivariate logistic regression models in age group 80– (N = 118).

Variables	n	Prevalence of HBP (%)	OR (95% CI)^a^	OR (95% CI)^b^
Gender: Female	79	49.4	–	1.000(reference)
Male	39	59.0	–	0.535(0.128, 2.237)
Body weight: normal weight	65	53.8	1.000 (reference)	1.000(reference)
Leanness	43	51.2	0.917(0.422, 1.992)	0.872(0.363, 2.096)
Overweight	7	42.9	0.620(0.127, 3.023)	0.407(0.063, 2.630)
Obesity	3	66.7	1.987(0.169, 23.372)	1.656(0.120, 22.941)
Education level: No school	76	48.7	1.000 (reference)	1.000(reference)
Primary school	9	66.7	1.869(0.405, 8.628)	3.417(0.642, 18.177)
Junior high school	23	56.5	1.139 (0.349, 3.717)	1.796(0.460, 7.013)
Senior high school	7	57.1	1.099 (0.176, 6.862)	1.586(0.179, 14.041)
Junior college and above	3	66.7	1.986 (0.170, 23.163)	c
Diabetes mellitus: no	117	53.0	1.000 (reference)	1.000(reference)
Yes	1	0	c	c
Cigarette smoking: no	85	49.4	1.000 (reference)	1.000(reference)
Smoking	33	60.6	1.390(0.456, 4.240)	1.414(0.408, 4.899)
Alcohol consumption: no	99	46.5	1.000 (reference)	1.000(reference)
Yes	19	84.2	5.992(1.637, 21.935)	8.407(2.161, 32.707)
Family history of HBP: no	84	45.2	1.000 (reference)	1.000(reference)
Yes	34	70.6	2.788(1.165, 6.672)	4.110(1.557, 10.848)

95%CI, 95% confidence interval; HBP, high blood pressure; OR, odds ratio;

a Adjusted for gender only; ^b^ Adjusted for all other variables in the table; ^c^ the value is not applicable because the number is too small.

Compared with participants without schooling, those who finished senior high school, junior college or above were less likely to suffer from hypertension(OR<1.0). This result was not observed among the subjects who only finished primary school or junior high school. In the mean time, diabetes mellitus was not associated with hypertension, according to the result of the analysis.

## Discussion

The overall prevalence of hypertension among Bai ethnic group aged 50 or more in rural southwest China in the study was higher than those among people from other areas in China [3], [4], [6], [19], Europe [20], and USA [5], [21]. It was lower than those in populations from Thailand [22] and South Korea [23](Table 8). However, if age groups are taken into account, the prevalence rates were lower than those in individuals between 55 and 64 years old [6]. Similar phenomena were observed in both within and outside China [4], [5].

**Table 8:** Comparison of prevalence, awareness, treatment, and control of hypertension in China and other countries.

Study (author/s)	Project Site	Sample (n)	Year of Study	Age	Hypertension			
					Prevalencê	Awareness	Treatment	Control
The present study	Rural Southwest China	2,133	2010	≥50	40.0%	28.4%	86.7%^a^(24.6%^b^)	30.0%^c^(7.5%^d^)
Wu Y et al [3]	Rural and urban China	141,892	2002	≥18	18%	25%	80% a (20%^b^)	24% c (5% d)
Yang J et al [6]	Rural Eastern China	8,359 18,922 20,167	1991 20022007	35–74	20.4% 24.5% 30.6%	15.0% 49.0% 52.4%	8.9% b 38.8% b 38.3% b	3.3% d 6.3% d 7.2% d
Meng XJ et al [19]	Urban Northeast China	25,196	2010	18–74	28.7%	42.9%	65.7% a (28.2% b)	12.9% c (3.7% d)
Huang J et al [4]	Rural Southeast China	5,350	*	*	36.09%	28.85%	*	*
Porapakkham Y et al [22]	Thailand	19,374	2004	≥60	51.1%	43.9%	36.1% a (8.5% b)	10.6% c (1.7% d)
Lee HS et al [23]	Rural South Korea	6,388	2005–2006	≥40	43.8%	60.1%	91.7% a (55.1% b)	27.2% c (16.1% d)
Costanzo S et al [20]	London(England) Limburg(Belgium) Abruzzo(Italy)	1,604	2001–2003	26–65	20.8% 23.6% 28.8%	44%	32.1% a 42.5% a 43.5% a	47.7% c 43% c 42% c
Angell SY et al [5]	New York City, USA	1,975	2004	≥20	25.6%	75.0%	62.5% a	43.6% c
Burt VL et al [21]	USA	9,901	1988–1991	≥18	24%	69%	53% a	45% c(24% d)

? the diagnostic criteria included SBP≥140, DBP≥90, and self-reported HBP treatment.

a the proportion of hypertensive patients who took antihypertensive medications among the hypertensive patients who were aware of their hypertension.

b the proportion of hypertensive patients who took antihypertensive medications among all the hypertensive patients.

c the proportion of hypertensive patients who had their HBP controlled among the hypertensive patients receiving medical treatment for hypertension.

d the proportion of hypertensive patients who had their HBP controlled among all the hypertensive patients.

* denotes the information was not available.

The fact that the participants in this study were older than those in other studies mentioned above might be helpful to explain why the prevalence of hypertension was higher in this study than those in the investigations focusing on both young and old participants. Other possible reasons for this result of high prevalence of HBP may include the following factors: first of all, the high altitude of the habitat for the Bai ethnic group, which is about 2000 meters above sea level; secondly, the Bai group have a preference for preserved foods, which implies that they may intake more salt than other such ethnic groups without this preference as Dai ethnic group in the same province; and thirdly, they have a preference for Rushan – a dairy product in Dali, which means lipid intakes may be higher in Bai group than in such ethnic groups as Dai, Yi in the same area.

The percentage of hypertensive participants who were aware of their condition was lower as compared to the data from other Asian countries [22], [23], Europe [20], the United States [5], [21], and even the eastern and northeast parts within China [6], [19], although it was higher than those in the Chinese population as a whole [3].

There are some possible reasons for the low awareness percentage, one of which might be poor publicity of hypertension related management information in the area. The other reason would be a popular idea among the rural communities in Dali, which went that “do not go to see a doctor until you can not tolerate a health problem any longer”, and this implied that there might be a big proportion of the hypertensive patients who were not diagnosed at the early stage.

However, the proportion of those with HBP who received antihypertensive medication regimens among those aware of their condition appeared higher (86.7%) in the investigation than the proportions in Thailand (36.1%) [22] and northeast China (65.7%) [19], but lower than that in Korea (91.7%) [23]. However, the proportion is lower (49.0%) if the Luobuma, regarded as an antihypertensive medication in China but not in the western world, is removed from the list of medication for hypertension. The higher this proportion, the more likelihood for the hypertensive subjects to accept medical interventions. In this regard, hypertension screening programs should be regularly carried out in order for the hypertensive participants to be aware of their condition as early as possible for early medical interventions. In addition, health education projects may also be an option to share with the communities about the importance of going to the health center for regular check-up, including the measurement of blood pressure.

The percentage of hypertensive participants who had controlled their BP was observed higher in the survey than relevant proportions in the studies in Thailand [22], and in northeast China [19], [24], but lower than those in Europe [20] and in the United States [21]. BP control rate is a very important indicator. The low rate of BP control in the Bai ethnic population might result from one of the main criteria of many local people for their selection of medications, which was the price other than the effect of the medications when there was not enough information available about the effectiveness of the regimens. Of all the hypertensive patients who took medications for their HBP in the study, 43.4% (96/221) took Luobuma which is a traditional Chinese patent medicine easily available and not expensive, in order to control their high blood pressure. The medicine is also known as Apocynum venetum leaf extract whose efficacy is relatively mild, which implies the goal of antihypertension might not be guaranteed. Additionally, lack of information on the right management of hypertension might be another cause of the low control rate. For instance, some patients did not know that they needed to take antihypertensive medications in their whole lives. Instead, they believed that they could stop the medication regimens when their HP was checked and found “normal”. Therefore, it is crucial to promote health education to enable the communities to realise the importance of being compliant in medical treatments and go for regular check-ups.

Furthermore, risk factors for hypertension identified in the study include overweight/obesity, cigarette smoking, alcohol drinking, family history of HBP, and older age. Overweight and obese participants were more likely to have hypertension than normal-weight ones, while leanness was a protective factor for hypertension, which was also seen in the results of studies in Europe [20], eastern China [6], and northeast China [19]. Odds Ratios for overweight vs normal-weight subjects were 1.591(95%CI:1.211, 2.091) in this study, 2.13(95%CI:1.99, 2.28) in the eastern China [6], and 2.00(95%CI:1.80, 2.23) in the northeast China [19].

Smoking and alcohol drinking were also identified as risk factors of HBP, also seen in other studies on populations in eastern China [3], while family history of HBP was another risk factor, also found in northeast China [19], according to the analysis of the present study. Odds Ratios for smoking vs non-smoking participants were 1.415(95%CI:1.128, 1.774) in this study, 1.21(95%CI:1.02, 1.53) in the eastern China [6], and 1.05(95%CI:0.89, 1.25) in the northeast China [19]. Odds Ratios for alcohol drinking vs non-alcohol drinking people were 1.656(95%CI:1.290, 2.125) in this study, 1.19(95%CI:1.07, 1.31) in the eastern China [6], and 1.40 (95%CI:1.18, 1.67) in the northeast China [19]. Older age was also a risk of having HBP, which was found in other studies as well [6], [19], [20].

Additionally, education levels were found correlated to HBP. Finishing senior high school or above was identified as a protective factor for HBP, whereas finishing junior high school or below was not. A possible explanation for this result might be that, the better educated the people are, the more information about how to deal with HBP they have access to. However, diabetes mellitus did not show associated with hypertension based on the results of the survey.

Based on the results of this study, the hypertensive patients might be aware of the importance of the antihypertensive treatment, but did not know the right way to achieve their goal of keeping their blood pressures under control. As a result, it is suggested that community and/or clinic-based antihypertension programs be designed and implemented in the Bai ethnic communities in order to regularly check their BP and monitor the effects of antihypertensive medications for the people with HBP.

Finally, the limitations of the study should be noted. First of all, it was indicated that BP can decrease substantially over successive visits [25], while recommendations on hypertension require that the diagnosis of hypertension should be based on readings from several visits. As a result, the estimates of HBP in the present study, which were based on readings on a single day, may overestimate the prevalence of hypertension. Secondly, most of the participants did not allow us to take blood samples for lipids and glucose testing, according to their tradition. Therefore, the correlation between hypertension and diabetes mellitus was based merely on the analysis of a few blood samples and histories of diabetes millitus, which might be underestimated.

In conclusion, hypertension was found highly prevalent among Bai ethnic adults aged 50 and above in the rural southwest China (40.0%). The rate of awareness of hypertension was low (28.4%), while the treatment rate was also low (24.6%) with an even lower rate of controlled hypertension (7.5%). These results suggest that effective antihypertension programs be an urgent need for the Bai ethnic communities in terms of health education on hypertension management as well as hypertension screening, counseling, and treatment.
